# RNA-binding proteins regulate immune-related alternative splicing in inherited salt-losing tubulopathies

**DOI:** 10.1186/s13023-025-03972-1

**Published:** 2025-08-09

**Authors:** Fuhui Ma, Yanrong Ma, Mayinu Yusufu, Reziwanguli Wusiman, Shuqing Xing, Xiangxin Song, Suli Li, Yanying Guo

**Affiliations:** 1 Department of Endocrinology and Metabolism, People’s Hospital of Xinjiang Uygur Autonomous Region, Xinjiang Clinical Research Center for Diabetes, 91 Tianchi Road, Urumqi, Xinjiang 830001 China; 2 Xinjiang Key Laboratory, Cardiovascular Homeostasis and Regeneration Research, Urumqi, Xinjiang, 830001 China

**Keywords:** Inherited salt-losing tubulopathies, Hypokalemia, Immune, Transcriptomic, RNA-binding proteins, Alternative splicing events

## Abstract

**Background:**

Inherited salt-losing tubulopathies (SLT) are rare disorders caused by gene mutations that disrupt renal tubular ion transport. However, the molecular mechanisms underlying SLT pathogenesis remain unclear. This study aims to elucidate the functional genes and potential regulatory mechanisms associated with SLT.

**Methods:**

We established a study cohort comprising inherited SLT patients, age-matched patients with acquired hypokalemia, and healthy volunteers. Clinical characteristics were compared among the groups. RNA sequencing (RNA-seq) was performed to obtain transcriptomic profiles, followed by analysis of gene expression patterns and alternative splicing events (ASEs). Key findings were validated using RT-qPCR.

**Results:**

SLT patients exhibited a higher prevalence of recurrent viral infections (65%, *P* = 0.004) and autoimmune thyroid disorders (30%, *P* = 0.022) compared to healthy controls. RNA-seq analysis identified 2,611 differentially expressed genes (DEGs) in SLT patients, including 1,236 upregulated and 1,375 downregulated genes. These DEGs were primarily enriched in innate immune responses and adaptive immunity pathways. Additionally, significant alterations in gene expression related to viral defense and stress responses were observed. Notably, we identified several RNA-binding proteins (RBPs) that may contribute to SLT pathogenesis by regulating ASEs of immune-related genes.

**Conclusion:**

Our findings highlight the critical role of RBPs in SLT pathogenesis and provide novel insights into the immune profiles and gene expression dynamics in SLT. This study lays the foundation for future research into targeted therapies and personalized treatment strategies for SLT management.

**Supplementary Information:**

The online version contains supplementary material available at 10.1186/s13023-025-03972-1.

## Introduction

Inherited salt-losing tubulopathies (SLT) are a group of rare genetic disorders caused by mutations in genes encoding sodium or other ion transporters, leading to impaired sodium and chloride transport in the loop of Henle or distal tubules [1]. These defects result in reduced reabsorption of sodium and chloride ions, causing relative blood volume contraction, secondary hyperaldosteronism, and increased excretion of potassium and hydrogen ions in the collecting ducts. Consequently, patients develop hypokalemia, metabolic alkalosis, and normal blood pressure. SLT play a critical role in renal ion transport mechanisms, with Bartter syndrome (BS) and Gitelman syndrome (GS) being the two most common types. Both are rare autosomal recessive disorders characterized by chronic hypokalemia due to renal salt wasting. GS patients often exhibit hypomagnesemia due to chronic renal magnesium depletion. Common clinical manifestations include muscle weakness, cramps, and fatigue [2, 3].

Recent research has increasingly highlighted the association between SLT and autoimmune diseases. Multiple case reports have documented the coexistence of BS or GS with conditions such as Sjögren’s syndrome, adult-onset autoimmune diabetes, autoimmune thyroid disease, and vasculitis [4–10]. Studies have also shown that the immune function of SLT patients is in a hyperactive state, suggesting a potential link between SLT and immune dysregulation. For instance, a UK-based study involving 47 SLT patients with genotypes of BS, GS, and EAST syndromes demonstrated that the ionic environment in these patients impairs IL-17 immune activity. However, in vitro experiments showed that IL-17 responses could be reactivated by increasing extracellular ion concentrations [11]. These findings raise critical questions about the interplay between electrolyte imbalances and immune dysregulation in SLT, underscoring the need for further investigation into the underlying mechanisms.

Electrolyte disturbances are known to exert substantial effects on immune regulation, particularly in the context of inflammatory and autoimmune processes. Sodium ions, for instance, play a critical role in modulating both innate and adaptive immune responses. Elevated sodium levels promote the activation of pro-inflammatory cell subtypes, such as Th17 cells and M1 macrophages, while suppressing regulatory T cells [12, 13]. Similarly, potassium ions and potassium channels are essential for regulating T cell proliferation, differentiation, and effector function, as well as monocyte/macrophage activity [14, 15]. Magnesium ions, on the other hand, are crucial for immunoglobulin synthesis, complement activation, and the regulation of phagocyte function and T lymphocyte maturation [16]. These findings indicate that chronic electrolyte depletion in SLT patients could underlie immune dysfunction, although this hypothesis requires further validation. SLT, as a unique model of chronic salt depletion, provide a valuable platform to investigate whether dysregulation of immune-related gene expression also contributes to disease mechanisms.

Based on this, our study enrolled patients with genetically confirmed SLT as the disease group, alongside individuals with acquired hypokalemia and healthy volunteers as controls. Using RNA-seq technology, we aimed to identify key genes and potential molecular mechanisms underlying immune dysfunction in SLT. This research is expected to provide new insights into the diagnosis and potential therapeutic targets for SLT, ultimately improving patient outcomes.

## Material and methods

### Participants

We enrolled 20 patients with genetically confirmed SLT(iSLTs group), 20 age-matched healthy controls (HC group), and 20 patients with acquired hypokalemia (C_hk group). The iSLTs group included 2 patients with BS and 18 with GS (Table S1). The C_hk group comprised patients with acquired hypokalemia, including 4 cases of primary aldosteronism, 8 cases of Graves’ disease, 5 cases of Cushing’s syndrome, 2 cases of diuretic-induced hypokalemia, and 1 case of renal tubular acidosis. This study was approved by the Ethics Committee of the People’s Hospital of Xinjiang Uygur Autonomous Region, China (No. KY2021052650). Ethical approval of the study protocol was obtained from the hospital in accordance with the Declaration of Helsinki, and written informed consent was obtained from all participants. Participants aged ≥ 18 years were asked to provide informed consent for themselves, while for individuals < 18 years, approval was obtained from their parents.

Given the rarity of genetically confirmed SLT (estimated prevalence: 1–10/100,000) [17, 18], our cohort size (*n* = 20/group) was constrained by case availability over a 3-year recruitment period. To address potential power limitations, we focused RNA-seq analysis on large-effect transcriptional alterations (|logFC|> 1, FDR < 0.05); post-hoc power analysis confirmed > 80% power (α = 0.05, d = 1.2) for primary RNA-seq endpoints. Data collection included anthropometric measurements, personal medical history, and biochemical tests.

### Genetic diagnosis

Peripheral blood samples (3 mL) were collected in tubes containing 50 mmol/L EDTA-2Na, and whole blood DNA were extracted by QIAamp Genome Extraction Kit (No. 51306, QIAGEN, Germany), and gene sequencing were entrusted to Beijing Macro & Micro- Test Bio-Tech Co.,Ltd. The exons were captured by the Roche Nimblegen SeqCap EZ ShareChoice XL custom microarray and sequenced on the Illumina sequencing platform, with target sequence coverage of at least 99%. The sequencing data were aligned to the human genome by BWA (0.7.12-r1039) software [19], and the mutation sites were annotated with dbSNP, Clinvar, ExAC, Thousand Genomes, and other databases using annovar [20] ($Date: 2015–06-17), and the suspected pathogenic mutations were analyzed according to the genetic variant classification system of ACMG (American College of Medical Genetics and Genomics). The suspected pathogenic mutations were graded according to the ACMG genetic variant grading system, and the unreported mutations or mutations of uncertain clinical significance were predicted by the software and annotated in the population database. The target sequences of suspected pathogenic mutations were subjected to PCR, and the sequencing results were verified using DNASTAR’s SeqMan subroutine software.

### RNA extraction and sequencing

Total RNA was treated with RQ1 DNase (Promega) to remove DNA. The quality and quantity of the purified RNA were determined by measuring the absorbance at 260 nm/280 nm (A260/A280) using smartspec plus (BioRad). RNA integrity was further verified by 1.5% agarose gel electrophoresis. For each sample, 1 μg of total RNA was used for RNA-seq library preparation. mRNAs were captured by VAHTS mRNA capture Beads (Vazyme, N401).The purified RNA was treated with RQ1 DNase (Promega) to remove DNA before used for directional VAHTS Universal V8 RNA-seq Library Prep Kit for Illumina (NR605) Polyadenylated mRNAs were purified and fragmented. Fragmented mRNAs were converted into double strand cDNA. Following end repair and A tailing, the DNAs were ligated to Adaptor (N323). After purification of ligation product and size fractioning to 300-500bps, the ligated products were amplified and purified, quantified and stored at -80℃ before sequencing. The strand marked with dUTP (the 2nd cDNA strand) is not amplified, allowing strand-specific sequencing. For high-throughput sequencing, the libraries were prepared following the manufacturer’s instructions and applied to Illumina Novaseq 6000 system for 150 nt paired-end sequencing.

### RNA-seq raw data clean and alignment

Raw reads containing more than 2-N bases were first discarded. Then adaptors and low-quality bases were trimmed from raw sequencing reads using FASTX-Toolkit (Version 0.0.13). The short reads less than 16nt were also dropped. After that, clean reads were aligned to the GRCh38 genome by HISAT2 [21] allowing 4 mismatches. Uniquely mapped reads were used for gene reads number counting and FPKM calculation (fragments per kilobase of transcript per million fragments mapped) [22].

### Differentially expressed genes (DEGs) analysis

The R Bioconductor package DESeq2 [23] was utilized to screen out the DEGs. The P value for correction < 0.05 and fold change ≥ 2 or ≤ 0.5 were set as the cut-off criteria for identifying DEGs.

#### Identification of differentially expressed RNA-binding proteins (DE-RBPs)

Then expression profile of DE-RBPs were filtered out from all DEGs according a catalogue of a catalog of 2,141 RBPs retrieved from four previous reports [24–27].

#### Alternative splicing analysis

The alternative splicing events (ASEs) and regulated alternative splicing events (RASEs) between the samples were defined and quantified by using the ABLas pipeline as described previously [28]. In brief, ABLas detection of ten types of ASEs was based on the splice junction reads, including intron retention (IR), exon skipping (ES), alternative 5’ splice site (A5SS), alternative 3’splice site (A3SS), mutually exclusive exons (MXE), mutually exclusive 5’UTRs (5pMXE), mutually exclusive 3’UTRs (3pMXE), cassette exon, A3SS&ES and A5SS&ES. To assess statistical differential ASE, Student’s t-test was performed to evaluate the significance of the ratio alteration of AS events.

#### ASE analysis on immune-related genes

1793 immune-related genes were retrieved from the ImmPort database (https://www.immport.org/shared/genelists).

#### Co expression analysis

Co-expression analysis was performed for RBPs and ASEs. Meanwhile, Pearson correlation coefficient between RBP and ASE was calculated, RBP and ASE relationship pairs satisfying absolute value of correlation coefficient ≥ 0.6 and *P* value ≤ 0.01 were screened.

#### Functional enrichment analysis

To sort out functional categories of DEGs, Gene Ontology (GO) terms was identified using KOBAS 2.0 server [29]. Hyper geometric test and Benjamini–Hochberg false discovery rate (FDR) controlling procedure were used to define the enrichment of each term.

#### RT-qPCR

RT-qPCR was performed on the ABI QuantStudio 5, followed by denaturing at 95˚C for 10 min, 40 cycles of denaturing at 95˚C for 15 s and annealing and extension at 60˚C for 1 min. Each sample had three technical replicates. The concentration of each transcript was then normalized to GAPDH (glyceraldehyde-3-phosphate dehydrogenase) and mRNA level using 2-^ΔΔCT^ method to analysis [30]. Comparisons were performed with the two-way ANOVA or the paired Student’s t-test by using GraphPad Prism software (Version number8.0, San Diego, CA).

In addition, we verify that the ASEs using RT-qPCR assay: the principle of primer design is that the one primer is designed at the boundary-spanning position of the constitutive exon and the alternative exon of the transcript, and the other primer is designed at the constitutive exon of the transcript; The boundary-spanning primer is designed on the template transcript to detect the splicing of the template transcript. And the detection of alternative spliced transcripts is similar. To evaluate the difference of alternative splicing, we calculated the ratio of the alternative splicing transcript/(alternative splicing transcript + template transcript) and compare the difference of ratios between different samples.

### Statistics analysis

Demographic and clinical variables were reported as mean ± standard deviation (SD) for continuous variables and frequency (percentage) for categorical variables. After checking the distribution of continuous variables with a normality test, we used Student’s t-test to compare continuous variables if normal distributions were not rejected, and Mann–Whitney U test if normal distributions were rejected. Pearson’s chi-square test was used to compare categorical data. Two-sided Fisher’s exact probability test was used when 2 or more minimum theoretical frequencies < 5 in the 2 × 2 table. All statistical analyses were performed using R, version 4.2.1 (R Programming). *P* values < 0.05 were considered statistically significant, and *P* values < 0.01 were considered highly significant.

## Results

### Comparative analysis of clinical characteristics in iSLTs, C_hk, and HC cohorts

The iSLTs cohort had a mean age of 36.75 ± 12.98 years, with 8 patients (40%) being female. Compared to both the C_hk and HC groups, iSLT patients exhibited significantly lower serum magnesium levels (*P* < 0.001), along with elevated C-reactive protein (*P* < 0.001) and plasma renin activity (*P* = 0.001 vs. *P* = 0.023). Additionally, fasting blood-glucose, aldosterone, erythrocyte sedimentation rate were higher in iSLT patients than in the HC group (all *P* < 0.05). Although systolic blood pressure was slightly lower in the iSLT group compared to controls, the difference was not statistically significant (*P* > 0.05). Table 1 demonstrates that iSLTs patients had a higher prevalence of recurrent viral infections (65%, *P* = 0.004) and autoimmune thyroid disease (30%, *P* = 0.022) than the HC group.

**Table 1:** Comparison of clinical characteristics in the iSLTs versus C_hk and HC

Characteristics	iSLTs *N* = 20	C_hk *N* = 20	HC *N* = 20	*P*-value (iSLTs vs. C_hk)	*P*-value (iSLTs vs. HC)
Age (year)	36.75 ± 12.98	38.39 ± 12.55	35.23 ± 10.29	0.338	0.257
Sex (n, female; %)	8 (40)	12 (60)	11 (55)	0.206	0.342
*Anthropometric factors*					
BMI(kg/m^2^)	21.32 ± 4.04	21.82 ± 3.75	23.03 ± 3.14	0.664	0.238
SBP(mmHg)	112.87 ± 15.24	114.02 ± 21.33	118.02 ± 12.50	0.377	0.503
DBP(mmHg)	72.54 ± 12.53	70.72 ± 11.64	73.54 ± 9.65	0.658	0.557
*Biochemical data*					
FBG(mmol/L)	5.26 ± 0.92	5.33 ± 1.43	4.55 ± 1.15	0.465	0.046*
HbA1C(%)	5.68 ± 0.86	5.76 ± 0.94	5.35 ± 0.74	0.432	0.124
TG(mmol/L)	2.27(1.63,3.28)	2.23(1.58,3.19)	2.08(1.12,2.97)	0.788	0.456
TC(mmol/L)	4.55 ± 1.21	4.87 ± 1.32	4.41 ± 0.78	0.512	0.664
LDL-C(mmol/L)	2.82 ± 0.79	2.86 ± 0.89	2.55 ± 0.64	0.326	0.229
eGFR(mL/min/1.73 m^2^)	115.10 ± 12.60	116.22 ± 13.55	112.00 ± 15.36	0.530	0.463
Na(mmol/L)	136.83 ± 10.33	139.37 ± 12.46	138.56 ± 11.79	0.398	0.467
K(mmol/L)	2.83 ± 0.33	3.14 ± 0.28	4.23 ± 0.34	0.473	< 0.001***
Mg(mmol/L)	0.55 ± 0.19	0.87 ± 0.25	0.89 ± 0.12	< 0.001***	< 0.001***
PH	7.49 ± 1.26	7.45 ± 1.35	7.37 ± 1.19	0.469	0.108
Plasma renin activity (ng/mL.h)	3.97 ± 0.97	0.49 ± 0.12	1.23 ± 0.67	0.006**	0.023*
Aldosterone (ng/dL)	20.23 ± 10.26	19.27 ± 9.95	10.18 ± 4.36	0.378	0.017*
ESR(mm/h)	15.58 ± 8.18	12.56 ± 9.31	9.00 ± 2.13	0.156	0.012*
CRP(mg/L)	10.93(5.90,19.26)	3.97(2.50,32.22)	0.45(0.21,1.16)	< 0.001***	< 0.001***
*Questionnaire data*					
Bacterial infection	7 (35)	6(30)	5 (25)	0.736	0.490
Fungal infection	3 (15)	2 (10)	1 (5)	1.000	0.605
Recurrent viral infection	13 (65)	8 (40)	4 (20)	0.113	0.004**
Allergic disease	3 (15)	2 (10)	2 (10)	1.000	1.000
Diabetes or IGT	4 (20)	5 (25)	0	1.000	0.106
Autoimmune thyroid disease	6 (30)	7 (35)	0	0.736	0.022*

BMI: body mass index; SBP: systolic blood pressure; DBP: diastolic blood pressure; FBG: fasting blood-glucose; TG: triglyceride; TC: total cholesterol; LDL-C: low density lipoprotein cholesterol; eGFR: estimated glomerular filtration rate; ESR: erythrocyte sedimentation rate; CRP:C-reactive protein; IGT: impaired glucose tolerance. ***P < 0.001, **P < 0.01, *P < 0.05

### Transcriptome analysis reveals immune dysregulation in iSLTs

**Fig. 1:**
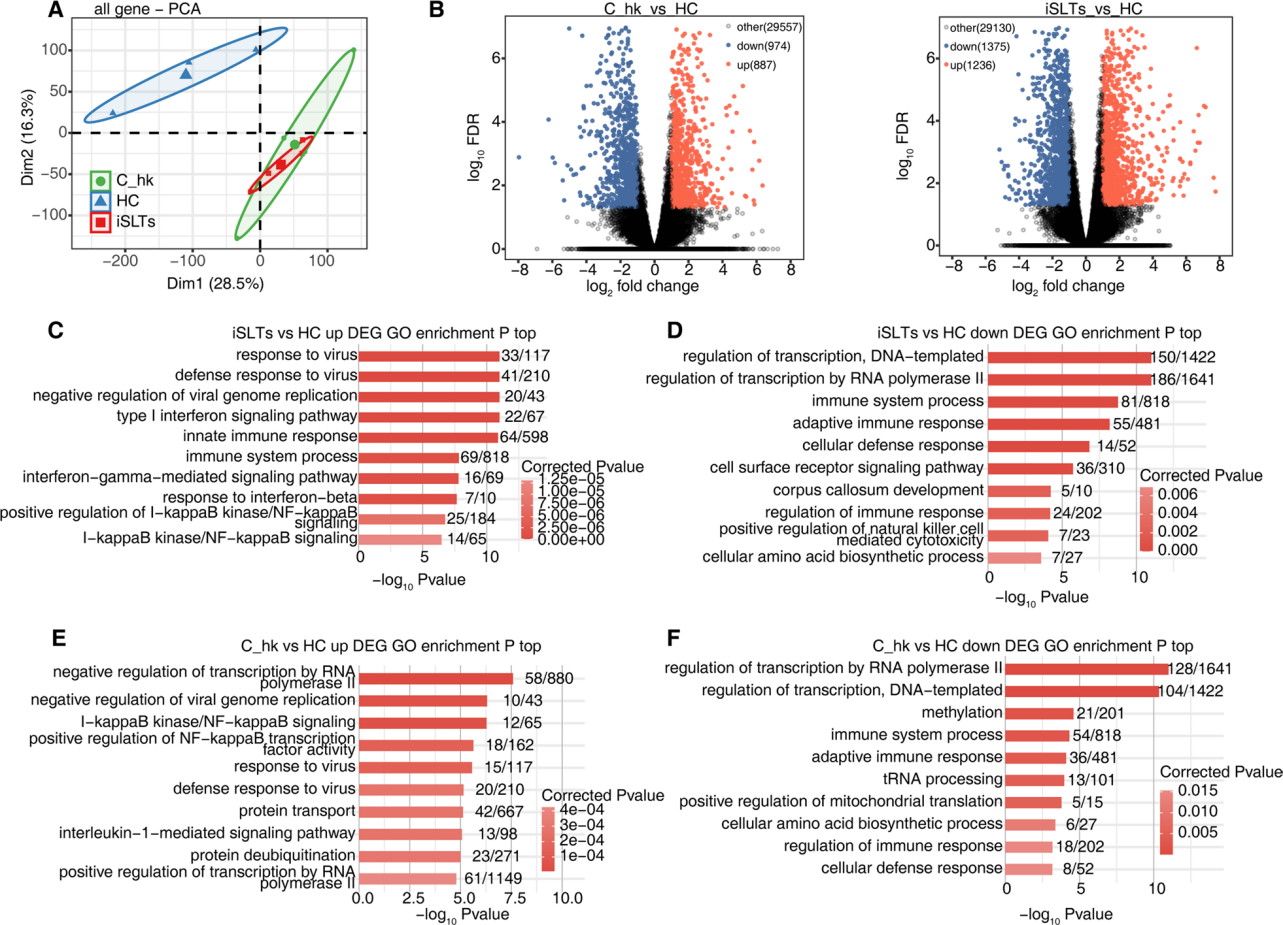
Transcriptome differences between iSLTs, C_hk and HC populations. **A** PCA plot of samples after normalizing all genes expression levels. The ellipse for each group is the confidence ellipse. **B** Volcano plot showing all DEGs between C_hk vs HC (left) and iSLTs vs HC (right) comparison group. Red indicates genes with upregulated expression. Blue indicates genes with downregulated expression. **C**–**F** Bar plot showing the most enriched GO results of up/down regulated genes between the different comparison groups

Principal component analysis (PCA) separated the iSLTs and C_hk groups from the healthy control (HC) group (Fig. 1A). DEG analysis identified 2,611 genes with significantly altered expression in the iSLTs group versus HC, including 1,236 upregulated and 1,375 downregulated genes (Fig. 1B). GO enrichment analysis revealed that the upregulated DEGs were predominantly enriched in pathways involving viral response and innate immunity, while the downregulated DEGs were primarily associated with transcription regulation and adaptive immunity **(**Fig. 1C-D). In contrast, 1,861 DEGs were identified between the C_hk and HC groups (887 upregulated, 974 downregulated, Fig. 1B). Upregulated DEGs in C_hk were mainly enriched in pathways including negative regulation of transcription by RNA polymerase II and I-kappaB kinase/NF-kappaB signaling pathway, whereas downregulated DEGs were primarily enriched in pathways related to adaptive immunity and immune regulation (Fig. 1E-F).

### Dynamic changes of DEG expression

**Fig. 2:**
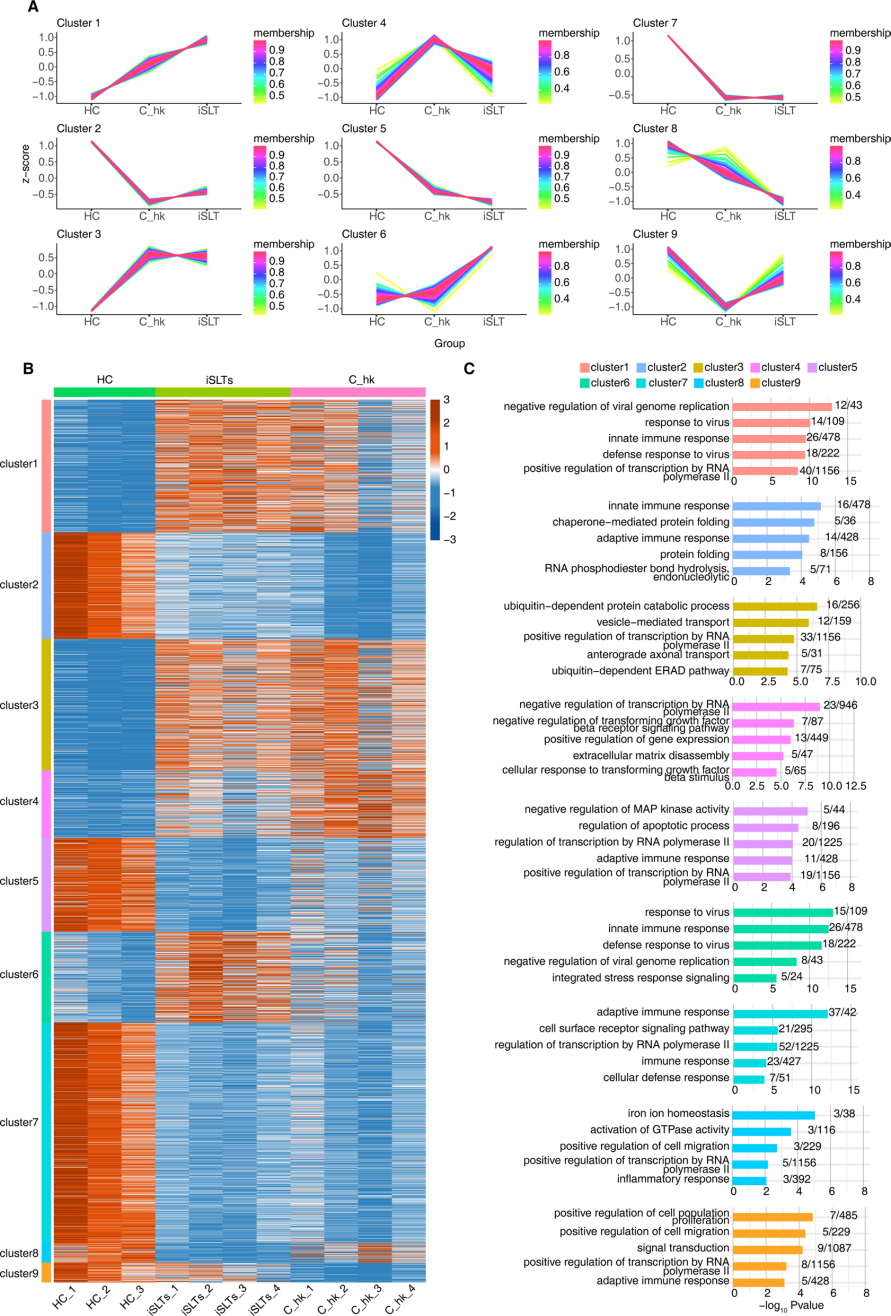
Dynamic analysis of DEGs. **A** DEGs of two comparison group (iSLT vs HC, C_hk vs HC) divided in nine clusters based on expression pattern. The expression trends of each cluster genes shown in line chart. **B** Hierarchical clustering heat map showing expression levels of all clustered DEGs. **C** The top 5 most enriched GO terms (biological process) were illustrated for DEGs in each cluster

Figure 2A presents DEG trend analysis performed using the TCseq package, grouping results into nine distinct clusters representing unique expression patterns. DEG expression in most clusters (1–3, 5, 7 and 8) remained similar between the iSLTs and C_hk groups, showing no significant alterations. This pattern may be associated with low potassium levels common to both groups. Notably, only cluster 6 exhibited markedly elevated expression specific to the iSLTs group, showing substantial differences compared to the C_hk group (Fig. 2B). This cluster likely represents genes directly linked to iSLTs pathogenesis. As anticipated, GO functional analysis of cluster 6 DEGs revealed significant enrichment in biological processes including virus response and innate immunity (Fig. 2C)..

### RBPs are associated with hypokalemia and SLT pathogenesis.

**Fig. 3:**
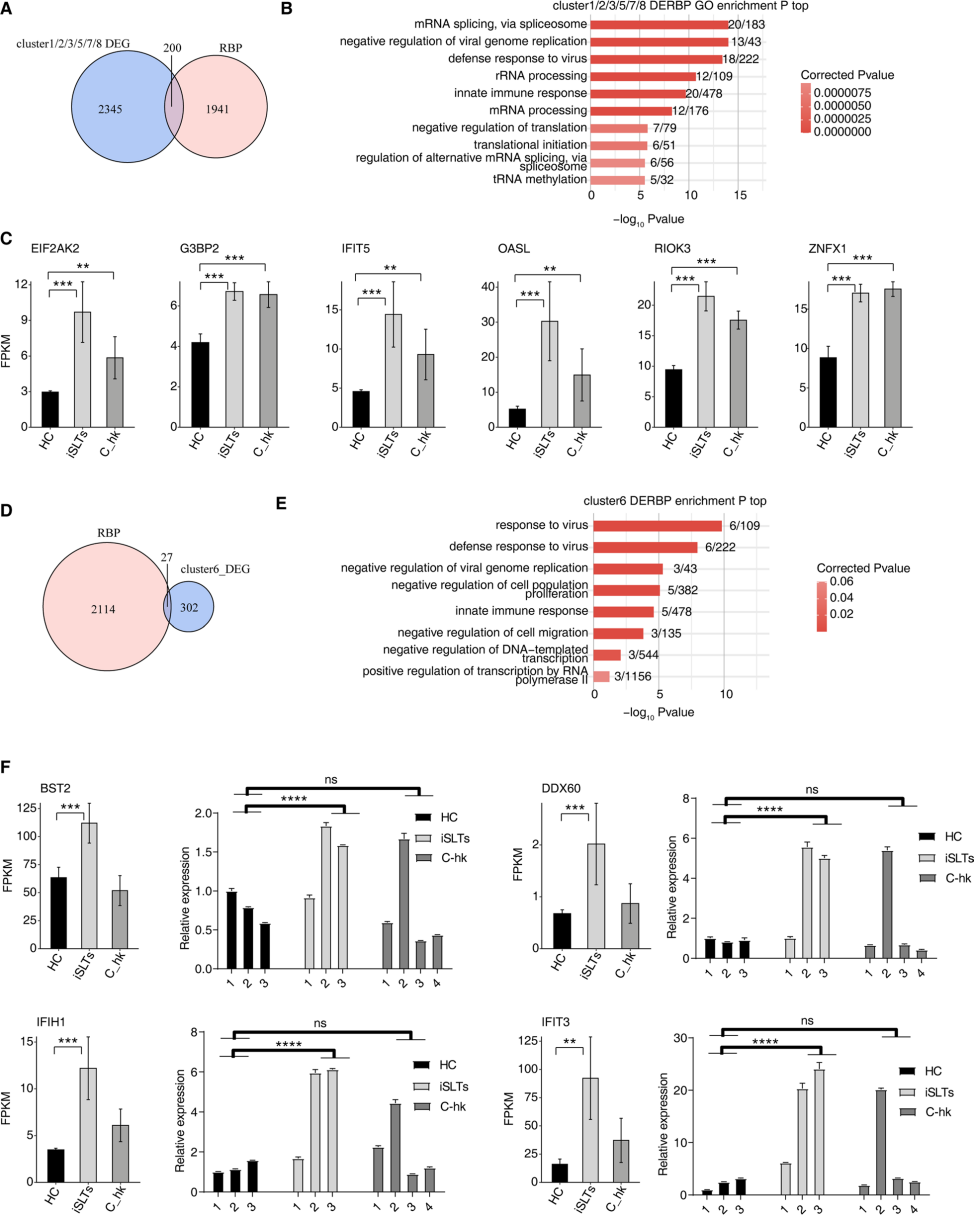
Identification of RBPs associated with salt-losing tubulopathies, hypokalemia disease controls and healthy population. **A** Venn diagram showing the overlapped genes between human RNA binding protein (RBP) genes and clustered gene set of cluster1, 2, 3, 5, 7 and 8. **B** Bar plot showing the most enriched GO results of overlapped RBPs in Fig. 3A. **C** Bar plot showing the expression pattern and statistical difference of selected DERBPs. Error bars represent mean ± SEM. ***P-value < 0.001, **P < 0.01. **D** Venn diagram showing the overlapped genes between human RBP genes and clustered gene set of cluster6. **E** Bar plot showing the most enriched GO results of overlapped RBPs in Fig. 3D. **F** Bar plot showing the expression pattern and statistical difference of selected DERBPs from RNA-seq and RT-qPCR. Error bars represent mean ± SEM. ***P-value < 0.001, **P < 0.01

RBPs regulate RNA metabolism post-transcriptionally and are implicated in genetic diseases [31], but their role in hypokalemia remains unclear. Intersecting known RBP genes with DEGs from clusters 1–3, 5, 7, and 8 identified 200 potentially potassium-associated DE-RBPs, as shown in Fig. 3A. These DE-RBPs were enriched in mRNA splicing, negative regulation of viral replication, viral defense, and innate immunity (Fig. 3B). Key RBPs (EIF2AK2, G3BP2, IFIT5, OASL, RIOK3 and ZNFX1) exhibited markedly elevated expression in the disease group (Fig. 3C). Analysis of cluster 6 identified 27 DE-RBPs (Fig. 3D) enriched in viral response/defense, negative regulation of viral replication/cell proliferation/migration, and innate immunity (Fig. 3E). Strikingly elevated expression of immune-related RBPs (IFIT3, BST2, DDX60, IFIH1) in iSLTs was confirmed by RT-qPCR (Fig. 3F).

### RBPs regulate alternative splicing of immune-related genes linked to disease progression

**Fig. 4:**
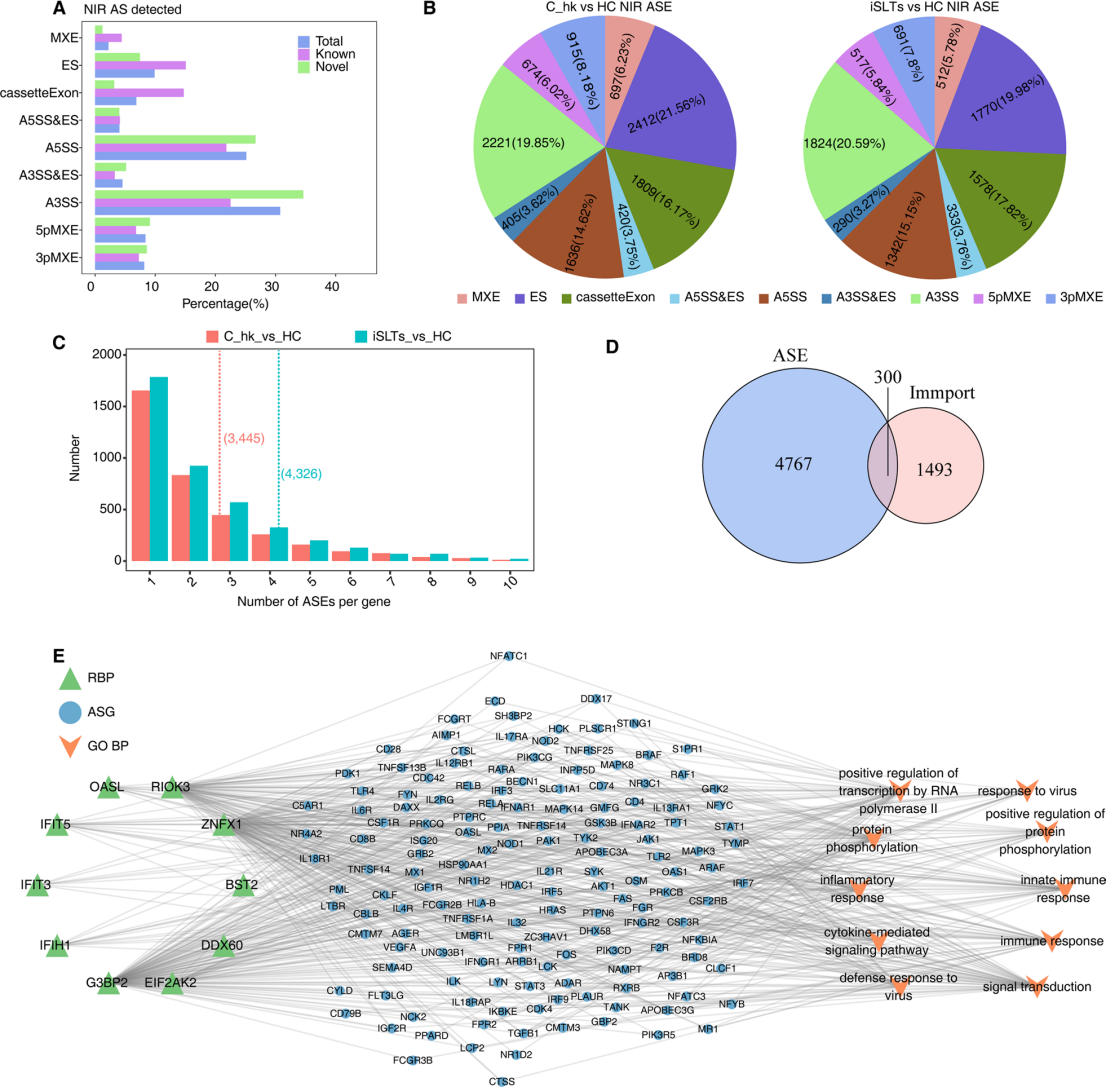
RBP-ASG regulation associated with hypokalemia or salt-losing tubulopathies. **A** The bar plot showing the percentage of all NIR alternative splicing events. **B** The pie plot showing the number of NIR significant ASEs in two comparison group C_hk vs HC (left) and iSLTs vs HC (right). **C** Bar plot showing the number of ASE per gene in two comparison group, median number marked with dashed line. Y axis: numbers of genes. X axis: numbers of ASE per gene. **D** Venn diagram showing the overlap of immune associated ASGs. **E** Co-expression network plot showing the regulatory network consisting of RBPs and immune-related ASGs and the top 10 most enriched GO terms. Co-expression relationship pairs were identified using a threshold of *P* value ≤ 0.01 and Pearson coefficient ≥ 0.6 or ≤ -0.6

Given the critical role of RBPs in AS, we identified significant differential ASEs across the groups: 8,857 in C_hk vs HC, 2,119 in iSLTs vs C_hk, and 11,189 in iSLTs vs HC. Focusing on nine non-intron retention (NIR) events, we found that A3SS and A5SS were predominant, followed by ES and cassette exon (Fig. 4A). Notably, ES and A3SS events were the primary ASEs altered in both C_hk and iSLTs (Fig. 4B). Additionally, many genes harbored multiple ASE types (Fig. 4C), GO analysis revealed that the corresponding ASE genes (ASGs) shared functions in RNA processing/splicing and viral processes (Fig. S1).

To investigate immune-related AS, we used the ImmPort database and identified 300 immune-associated ASGs (Fig. 4D). Co-expression analysis with differentially expressed RBPs suggested that RBPs influence immune responses by potentially regulating ASEs in these genes. Consistent with this, GO enrichment of co-expressed target ASGs confirmed their involvement in innate immunity, immune response, and inflammation (Fig. 4E).

**Fig. 5:**
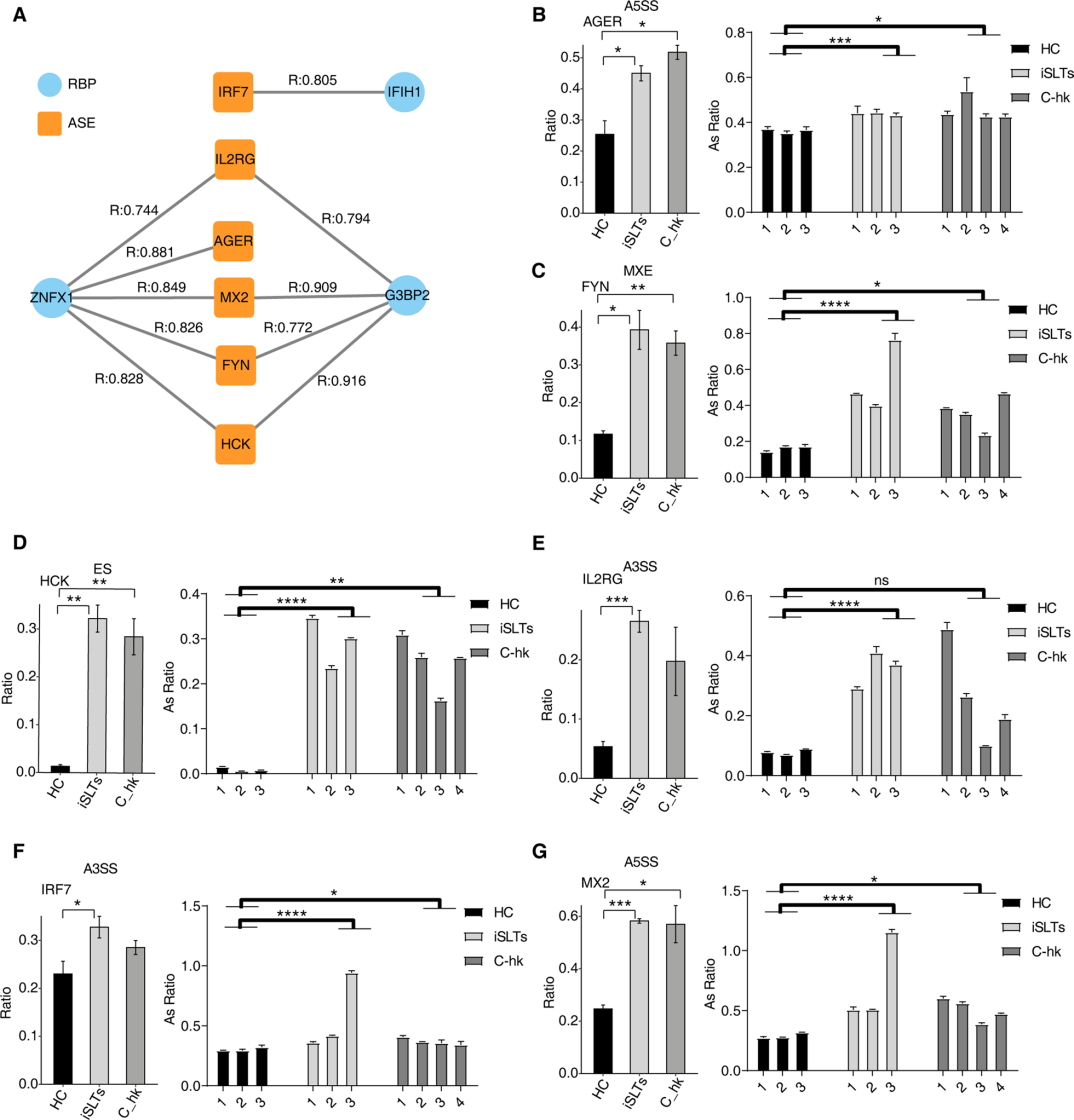
Identification of pathogenesis-related ASGs. **A** Regulatory networks between RBPs and pathogenic ASGs. **B**–**G** Bar plot showing the AS ratio and statistical difference of immune associated genes from RNA-seq and RT-qPCR. Error bars represent mean ± SEM. ***P < 0.001, **P < 0.01, *P < 0.05

Finally, we identified seven highly variable ASGs (MX2, IL17RA, FYN, AGER, IL2RG, HCK, IRF7) exhibiting strong positive or negative co-expression with four RBPs (G3BP2, ZNFX1, RIOK3, IFIH1) (Fig. 5A). RT-qPCR validation confirmed significant AS ratio changes for these genes, consistent with RNA-seq results (Fig. 5B-G).

## Discussion

Inherited salt-losing tubulopathy (SLT) imposes a substantial clinical and economic burden due to elevated healthcare costs and long-term care needs [32, 33]. While electrolyte imbalance explains cardinal symptoms (e.g., muscle cramps, fatigue, and dizziness), a subset of patients exhibit extra-renal manifestations—including recurrent childhood fever, autoimmune disorders, and viral infections [34, 35]—suggesting immune involvement. Our study defines three novel mechanistic dimensions of SLT pathogenesis: (1) Clinical immune dysregulation, characterized by strongly elevated inflammatory markers (CRP, ESR), hypomagnesemia, renin activation, and increased prevalence of recurrent viral infections and autoimmune thyroid disease; (2) Distinct transcriptomic signatures, with 2,611 differentially expressed genes (DEGs) versus controls and an iSLT-specific gene cluster (Cluster 6) enriched in antiviral/innate immunity pathways; (3) RBP-mediated splicing dysregulation, where 27 immune-related RNA-binding proteins (e.g., IFIT3, BST2) drive aberrant alternative splicing of key immune genes (e.g., MX2, IRF7), directly linking RBP dysfunction to inflammatory cascades in SLT. These findings establish immune dysregulation as a core pathogenic component, implicating RBP-directed splicing as a previously unrecognized disease mechanism.

The immune dysregulation observed in SLT patients may be linked to both ion metabolism disorders and altered gene expression. While chronic hypokalemia can induce low-grade inflammation, our data reveal iSLT-specific immune signatures. Notably, high sodium intake, a common feature in SLT management, has been shown to impair regulatory T cell (Treg) function, leading to increased pro-inflammatory cytokine production and a heightened risk of autoimmunity [36–38]. Additionally, prolonged hypokalemia affects the proliferation and differentiation of T cells and monocyte-macrophages, as well as the expression of pro-inflammatory cytokines such as IL-1β, IL-6, and TNF-α [39, 40]. Through RNA-seq analysis, we identified significant alterations in pathways related to viral infection and immune response in SLT patients, which align with their clinical manifestations. The differential expression of numerous genes highlights the molecular complexity of SLT. Importantly, we identified several RBPs that may serve as potential biomarkers for assessing disease severity and treatment response.

RBPs play pivotal roles in post-transcriptional gene regulation, influencing cellular responses to stress and infection [41]. Our findings suggest that RBPs associated with alternative splicing and immune regulation may represent a novel mechanism of pathogenesis in SLT [42]. Specifically, we identified four RBPs (G3BP2, ZNFX1, RIOK3, and IFIH1) and their potentially regulated alternatively spliced genes (ASGs) (MX2, IL17RA, FYN, AGER, IL2RG, HCK, and IRF7), which may be closely linked to the pathological changes in SLT. G3BP2, a key component of stress granules, is implicated in viral infection [43], while ZNFX1 is essential for initiating the type I interferon (IFN) response [44]. RIOK3 and IFIH1 are involved in IFN-dependent immune responses and play critical roles in sensing viral infections and activating antiviral cascades [45]. The ASGs regulated by these RBPs are potent immune regulators with antiviral and antimicrobial activities, suggesting that ASEs may contribute to disease progression. These splicing changes could impact not only immune responses but also other critical pathways involved in renal function and electrolyte balance.

As a rare genetic disorder, SLT provides a unique model for studying chronic salt depletion in vivo. This model is clinically significant for elucidating the underlying mechanisms or molecular targets associated with immune dysregulation, which could inform early interventions aimed at modulating immune function and reducing inflammation in SLT patients. The identification of RBPs and their regulated ASGs offers new insights into the molecular basis of SLT and highlights potential therapeutic targets. These findings not only lay the groundwork for the discovery of novel therapeutic strategies but also provide a foundation for developing biomarkers that could facilitate early diagnosis and personalized treatment approaches.

This study has several limitations that must be acknowledged when interpreting the findings. Firstly, While our cohort size (*n* = 20 per group) enabled robust detection of large-effect transcriptional alterations, it limits statistical power for subtle effects. Future multi-center collaborations are needed to validate generalizability. Secondly, although our integrated transcriptomic approach identifies compelling regulatory roles for RBPs in immune-related splicing, functional validation (e.g., RBP knockdown/overexpression in cell models) is warranted. This is also part of our planned follow-up research. Thirdly, the lack of in-depth clinical correlation analysis hinders the translation of these findings into clinical applications, such as the development of targeted therapies or biomarkers for improved patient management. Future studies should address these limitations by incorporating larger cohorts, experimental validation, and detailed clinical correlation analyses to ensure robust and clinically relevant conclusions.

## Conclusions

In conclusion, our research provides valuable insights into the gene expression profiles and immune characteristics of SLT patients, advancing our understanding of the disease’s underlying mechanisms. The identification of RBPs, along with significant alterations in immune responses and splicing events, highlights promising avenues for future exploration. These findings not only lay the groundwork for the discovery of novel therapeutic targets but also offer a new perspective for developing biomarkers that could facilitate early diagnosis and personalized treatment strategies. Continued investigation into these genetic and immunological factors will be crucial for advancing our knowledge and improving clinical outcomes for SLT patients.

## Supplementary Information

Supplementary material 1: (A)Bar plot showing the most enriched GO results of ASE related genes in C_hk vs HC comparison group. (B)Bar plot showing the most enriched GO results of ASE related genes in iSLTs vs HC comparison group.

Supplementary material 2

## Data Availability

The data generated during the current study are available from the corresponding author on reasonable request.

## Abbreviations

SLT: Inherited salt-losing tubulopathies
GS: Gitelman syndrome
BS: Bartter syndrome
ASEs: Alternative splicing events
RBPs: RNA-binding proteins
RNA-seq: RNA sequencing
DEGs: Differentially expressed genes
