# Supplementary figures and images for: RNA-binding proteins regulate immune-related alternative splicing in inherited salt-losing tubulopathies
Source: Orphanet J Rare Dis. 2025 Aug 9;20:416. doi: 10.1186/s13023-025-03972-1 (PMC12335119; doi:10.1186/s13023-025-03972-1)

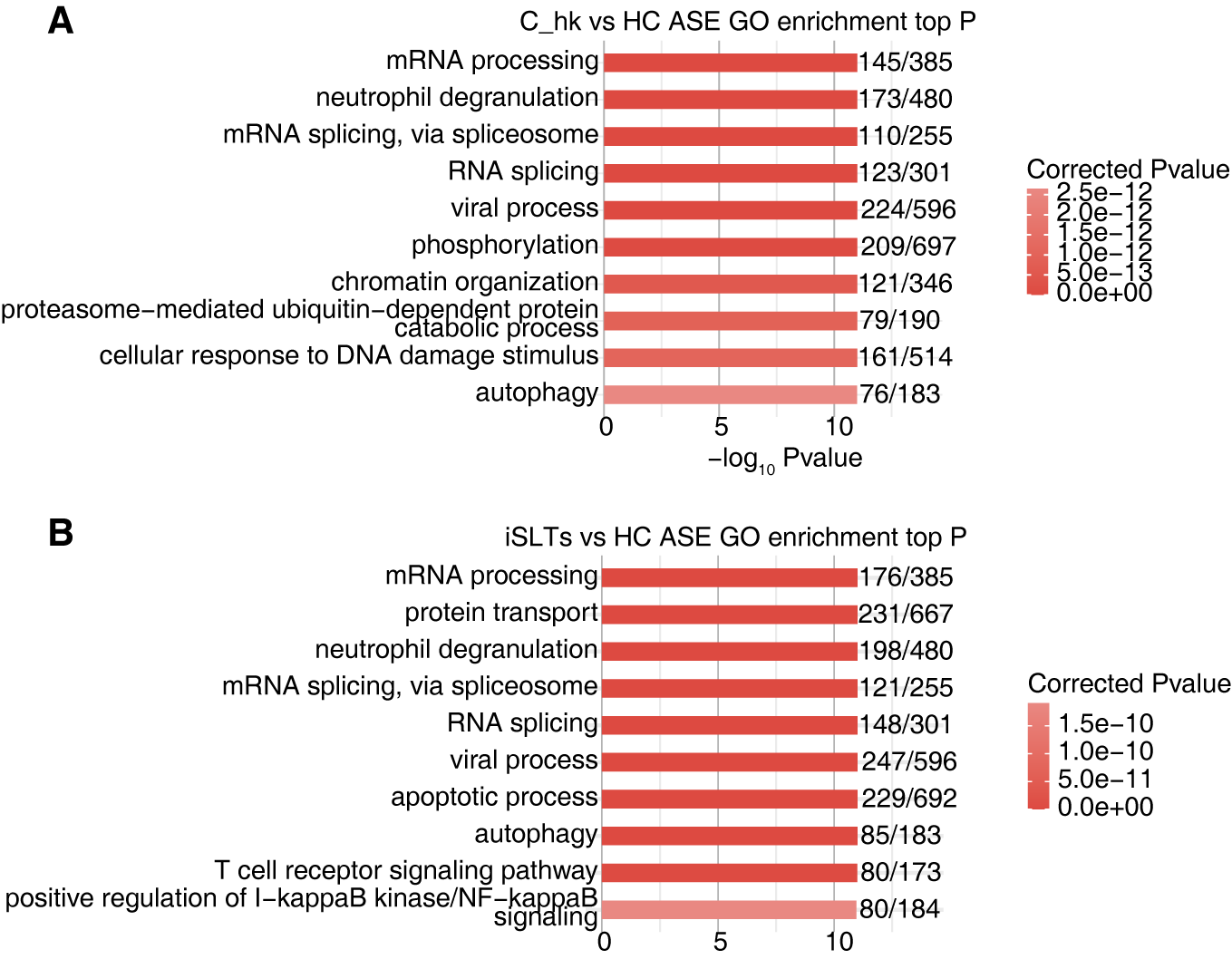

Supplement: Supplementary file 1 — Supplementary material 1: (A)Bar plot showing the most enriched GO results of ASE related genes in C_hk vs HC comparison group. (B)Bar plot showing the most enriched GO results of ASE related genes in iSLTs vs HC comparison group. [file 13023_2025_3972_MOESM1_ESM.tif]
