# Supplementary material for: RNA-binding proteins regulate immune-related alternative splicing in inherited salt-losing tubulopathies
Source: Orphanet J Rare Dis. 2025 Aug 9;20:416. doi: 10.1186/s13023-025-03972-1 (PMC12335119; doi:10.1186/s13023-025-03972-1)
Supplement: Supplementary file 2 — Supplementary material 2 [file 13023_2025_3972_MOESM2_ESM.docx]

**Table S1**.Demographic data and genotype of Salt-Losing Tubulopathy patients

| **Patients number** | **Gender** | **Age** | **Diagnosis** | **Gene affected** | **Nucleotide Change** | **Protein**  **change** | **Type of**  **mutation** | **Status** |
| --- | --- | --- | --- | --- | --- | --- | --- | --- |
| 1 | F | 17 | Gitelman | *SLC12A3* | c.T1288G;c.G2191A | p.C430G;p.G731R | Missense | Compound heterozygous |
| 2 | M | 54 | Gitelman | *SLC12A3* | c.G1456A | p.D486N | Missense | Heterozygous |
| 3 | M | 56 | Gitelman | *SLC12A3* | c.C179T;  c.T191A | p.T60M;p.V64E | Missense | Compound heterozygous |
| 4 | M | 35 | Gitelman | *SLC12A3* | c.C1963T | p.R655C | Missense | Homozygous |
| 5 | F | 41 | Gitelman | *SLC12A3* | c.C961T | p.R321W | Missense | Heterozygous |
| 6 | F | 30 | Gitelman | *SLC12A3* | c.G1387A | p.G463R | Missense | Heterozygous |
| 7 | M | 52 | Gitelman | *SLC12A3* | c.2660+2dupT | - | Frameshift | Homozygous |
| 8 | M | 51 | Gitelman | *SLC12A3* | c.G1456A | p.D486N | Missense | Heterozygous |
| 9 | M | 31 | Gitelman | *SLC12A3* | c.C1049T | p.S350L | Missense | Heterozygous |
| 10 | M | 28 | Gitelman | *SLC12A3* | c.G1964A | p.A655H | Missense | Homozygous |
| 11 | F | 44 | Gitelman | *SLC12A3* | c.1567+1G>A | - | Splicing | Heterozygous |
| 12 | F | 19 | Gitelman | *SLC12A3* | c.486-490delTACGGinsA;  c.C539G | p.T163Pfs*7;p.T180A | Frameshift | Compound heterozygous |
| 13 | F | 34 | Gitelman | *SLC12A3* | c.C179T;  c.C1077G | p.T60M;p.N359K | Missense | Compound heterozygous |
| 14 | F | 48 | Gitelman | *SLC12A3* | c.G1732A | p.V578M | Missense | Heterozygous |
| 15 | M | 16 | Gitelman | *SLC12A3* | c.C179T | p.T60M | Missense | Heterozygous |
| 16 | M | 67 | Gitelman | *SLC12A3* | c.G1456A | p.D486N | Missense | Homozygous |
| 17 | M | 20 | Gitelman | *SLC12A3* | c.T815C | p.L272P | Missense | Heterozygous |
| 18 | F | 21 | Gitelman | *SLC12A3* | c.C179T | p.T60M | Missense | Heterozygous |
| 19 | M | 16 | Bartter (type 3) | *CLCNKB* | exon 2-11 deletion | - | Deletion | Homozygous |
| 20 | M | 42 | Bartter (type 3) | *CLCNKB* | Whole gene deletion | - | Deletion | Homozygous |
